# Présentations inhabituelles d'un syndrome de Plummer-Vinson chez l'africain de race noire: à propos de deux observations

**DOI:** 10.11604/pamj.2014.19.145.3952

**Published:** 2014-10-14

**Authors:** Adama Berthé, Madoky Magatte Diop, Papa Souleymane Toure, Cheikh Tidiane Tall, Abdoul Fulgence Faye, Bernard Marcel Diop, Mamadou Mourtalla Ka

**Affiliations:** 1 Université de Thiès, UFR des Sciences de la Santé Ex l0^éme^ RIAOM, Thiès, Sénégal; 2 Service de Médecine Interne Centre Hospitalier Régional de Thiès, 1 Avenue Malick SY Prolongée, Thiès, Sénégal; 3 Université de Ziguinchor-UFR des Sciences de la Santé, Ziguinchor, Sénégal

**Keywords:** Plummer Vinson, dysphagie, brulure caustique, maladie de Biermer, Plummer Vinson, dysphagia, caustic burn, Biermer disease

## Abstract

Le syndrome de Plummer Vinson (SPV) est une affection rare caractérisée par une dysphagie cervicale associée à une anémie ferriprive et un anneau sur l’œsophage supérieur. Parfois, son mode de présentation inhabituelle peut faire errer le diagnostic. Le rétrécissement annulaire peut être de découverte fortuite lors d'une endoscopie digestive haute. Nous rapportons deux observations de syndrome de Plummer-Vinson chez des sujets de genre masculin et féminin. Celles-ci ont comme point commun une découverte fortuite lors d'une endoscopie digestive haute. La première observation concernait un garçon de 14 ans aux antécédents de brûlure caustique de l’œsophage dans l'enfance avec dysphagie haute passagère ne l'inquiétant pas depuis lors. Il était reçu en urgence pour une endoscopie digestive haute motivée par une dysphagie de survenue brutale secondaire à une prise d'aliment solide. L'examen clinique avait objectivé une chéilite angulaire. La biologie montrait un abaissement de la ferritinémie sans anémie. L'endoscopie avait mis en évidence un anneau circulaire franchi avec ressaut au niveau de la bouche de Killian. Elle avait également permis l'extraction d'un corps étranger à type de noyau de « pain de singe » mais la lumière de l’œsophage était infranchissable à partir du niveau d'arrêt. Le transit œsophagien montrait un ralentissement du produit de contraste au niveau de l'sophage cervical et thoracique sans lésions morphologiques. Dans la deuxième observation, il s'agissait d'une jeune femme de 35 ans adressée à l unité d'endoscopie digestive pour objectiver une gastrite atrophique sur une suspicion de la maladie de Biermer. La fibroscopie mettait alors en évidence, un rétrécissement annulaire infranchissable à 18 centimètres des arcades dentaires. La biologie montrait une anémie avec augmentation de la ferritinémie. Dans les deux cas, le traitement martial était systématique associé à des séances de dilatation aux bougies de Savary-Gilliar. L’évolution à court terme était favorable avec une amélioration de la dysphagie et une correction des anomalies biologiques. La dysphagie passagère peut faire errer le diagnostic d'un syndrome de Plummer-Vinson. Son association soit avec une sténose caustique de l sophage soit avec une maladie de Biermer est possible. Le risque de dégénérescence accru devrait motiver une surveillance rapprochée.

## Introduction

Le syndrome de Plummer-Vinson encore appelé syndrome de Kelly Patterson est caractérisé par une triade classique associant: une dysphagie, une anémie sidéropénique et la présence de membranes œsophagiennes. Il est de plus en plus décrit en Afrique au Sud du Sahara [1–4]. La dysphagie constitue le maitre symptôme pouvant le révéler. Cependant, certaines présentations inhabituelles sans dysphagie [5] peuvent faire errer le diagnostic. Toutefois, le risque de dégénérescence en carcinome épidermoïde au niveau du pharynx et de l′œsophage impose sa reconnaissance et sa prise en charge précoce. Nous rapportons deux observations de syndrome de Plummer-Vinson, de découverte fortuite, lors d'une endoscopie digestive haute réalisée respectivement au Centre Hospitalier Régional de Thiès et au Centre Hospitalier National de Pikine.

## Patient et observation

### Observation 1

SMD, âgé de 14 ans, est reçu en urgence pour une hyper sialorrhée et une dysphagie de survenue brutale secondaires à une prise d'aliments solides. Dans ses antécédents, il est retrouvé une notion de brûlure de l’œsophage secondaire à l'ingestion de soude caustique à l’âge de 4 ans, mis depuis lors sous diète semi-liquide et/ou liquide avec une très bonne évolution.

**Figure 1:**
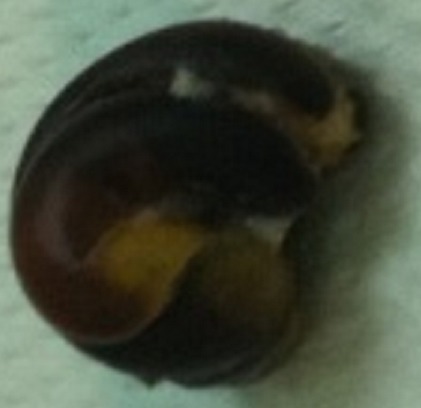
Noyau de « pain de singe »

**Figure 2:**
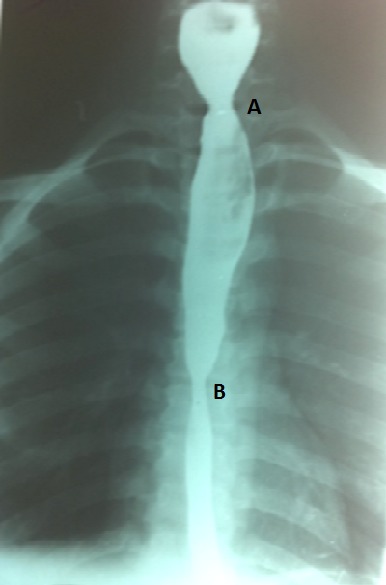
Rétrécissement de la bouche de Killian et de l'sophage thoracique (A et B)

Une endoscopie oeso-gastrique est effectuée le jour même et permet l'extraction d'un corps étranger: un noyau de la pulpe de « pain de singe » provenant du fruit du Baobab (Andasonia digitata L) (Figure 1). Toutefois dans les suites de l'extraction, l'exploration à la recherche d'une cause mécanique d'obstruction a montré que la lumière de l’œsophage était infranchissable à partir du niveau où le corps étranger était enclavé à cause de séquelles de brûlure. La muqueuse œsophagienne ne présentant aucune autre anomalie macroscopique. Cette endoscopie a aussi permis, la découverte fortuite d'un anneau au niveau de la bouche œsophagienne de Killian, ressenti par un ressaut lors du franchissement. Ainsi, le transit œsophagien montrait un ralentissement du produit de contraste au niveau de l’œsophage cervical et thoracique sans lésions morphologiques visibles (Figure 2).

Devant des signes cliniques suggérant une carence martiale du fait de la présence d'une chéilite angulaire, l'hypothèse d'un Plummer Vinson a été évoquée. Sur le plan biologique, l'hémogramme a montré un nombre de globules blancs à 3.300/mm^3^, avec des globules rouges à 4 millions/mm^3^ et un taux d'hémoglobine à 13,5g/dl pour un volume globulaire moyen à 83 fl (normale: 80-95 fl) et une concentration corpusculaire moyenne en hémoglobine à 33% (normale: 32-35%). La ferritinémie était basse à 17 ng/ml (normale 20-150 ng/ml). Comme traitement, le patient a reçu du sulfate ferreux à raison de 100 mg par jour pendant trois mois. Il a ensuite bénéficié de plusieurs séances de dilatations de l’œsophage à l'aide de bougies de Savary-Gilliar. L’évolution avec cette prise en charge, a été favorable avec disparition de la dysphagie.

### Observation 2

**Figure 3:**
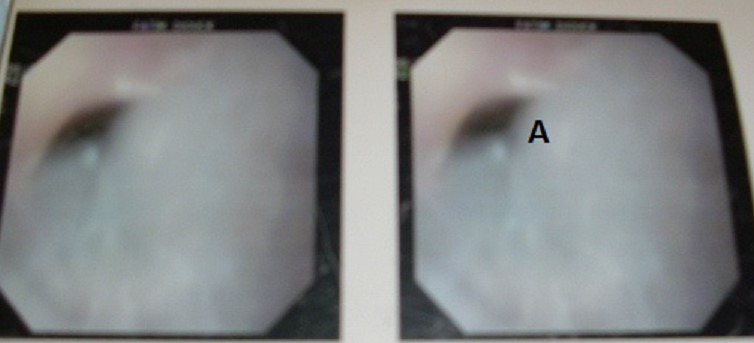
Membrane semi-lunaire sténosant la lumière de l’œsophage, à 18 cm des arcades dentaires (A)

Le 14 Avril 2010, Madame FD 35 ans était adressée à l'unité d'endoscopie digestive du CHN de Pikine, pour objectiver une atrophie gastrique sur une suspicion de maladie de Biermer. La fibroscopie mettait alors en évidence, un rétrécissement annulaire infranchissable à 18 centimètres des arcades dentaires (Figure 3).

Elle était admise depuis un mois au service de Médecine Interne du même hôpital, pour une anémie profonde. Le début de sa maladie remontait au mois de Novembre 2009, marquée par l'apparition d'une lombalgie intense à type de brûlure, d'irradiation descendante le long des membres inférieurs avec une notion d'anesthésie de la plante des pieds. Cette douleur était associée à des fourmillements. La patiente signalait aussi une dyspnée lors des efforts de la vie quotidienne et une coloration noirâtre acquise palmoplantaire. Mme D. se plaignait également de rectorragies intermittentes survenant lors de la défécation, le tout évoluant depuis 6 mois. Dans ses antécédents, elle avait été hospitalisée en Décembre 2009 et en Février 2010, pour une anémie profonde prise en charge à chaque fois par transfusions de sang total.

Elle était depuis lors, traitée par du fer à raison de 100mg par jour. Madame F D présentait aussi une dysphagie épisodique surtout lors de la prise d'aliments solides, mais qu'elle n'avait pas signalé avant l'endoscopie parce que la jugeant banale. La patiente n'est pas alcoolo-tabagique, n'a jamais été opérée et ses antécédents familiaux sont sans particularités. A l'examen on notait: un assez bon état général, des muqueuses pâles, une langue dépapillée; un abdomen souple sans hépato splénomégalie ni ascite; un prolapsus hémorroïdaire réductible.

A la biologie on notait: une pan cytopénie arégénérative, avec une leuconeutropénie (Globules blancs à 2200/mm^3^, Polynucléaires neutrophiles à 850/mm^3^) une thrombopénie modérée (à 146000 plaquettes/), une anémie à 3,9g/dl d'hémoglobine, macrocytaire (VGM à 119 fl), un taux de réticulocytes à 36750. La fonction rénale était normale avec une azotémie à 0,09 g/l, une créatininémie à 5mg/l. On notait une légère élévation des transaminases portant sur les ASAT (à 2 fois la normale) avec des ALAT dans les limites de la normale, à 16UI/l. L'ionogramme sanguin était normal. Le taux de Fer sérique était à la limite inférieure de la normale soit 65,59 microgramme /dl (pour une normale entre 65 et 175), avec un taux de Ferritine à 461,36 ng/ml (Normal entre 20et 150). Il n'y avait pas de syndrome inflammatoire. L'immunologie avait permis de détecter la présence d'Anticorps Anti-Facteurs Intrinsèque (U0607411 Laboratoire CERBA). La vitamine B12 sérique n'avait pas été dosée. L'endoscopie basse montrait des hémorroïdes au stade 2 avec des stigmates d'hémorragie. Comme traitement, La malade avait reçu de la cyanocobalamine à raison de 1000mg par jour pendant une semaine puis 1000mg par mois. L’évolution a été marquée par un passage du taux d'hémoglobine à 12g/dl avec un VGM à 92 fl, un taux de globules blancs à 4100/^3^ dont 2000 polynucléaires neutrophiles, un taux de Plaquettes à 190000. La malade était alors adressée à l'unité d'endoscopie interventionnelle du CHU Le Dantec, pour des dilatations aux bougies de Savary-Gilliar.

## Discussion

La prévalence du syndrome de Plummer-Vinson n'est pas bien établie. Celle-ci, s'explique par la rareté des publications mondiales sur le sujet. Cette affection était rarement rapportée en Afrique au Sud du Sahara jusqu’à une période récente [1–4]. Cette rareté contraste avec un contexte africain où la carence en fer et la malnutrition sont fréquentes. Nos observations semblent atypiques par leurs modes de présentations inhabituelles. En effet, le premier cas était découvert fortuitement lors de l'extraction d'un corps étranger enclavé dans l’œsophage thoracique, tandis que l'autre au cours du suivi d'une maladie de Biermer. Cette affection est l'apanage de la femme jeune d’âge mûr [2, 6] comme dans notre deuxième observation (35 ans). Toutefois, ce syndrome peut survenir chez l'enfant (observation 1) ou l'adolescent [7, 8].

Sur le plan clinique, la dysphagie constitue le maitre symptôme. Elle est généralement indolore et intermittente, limitée aux solides. Chez nos patients la dysphagie était présente. Elle était de siège cervicale haute, capricieuse (observation 2), passagère et tolérée jusqu’à la survenue brutale d'un arrêt en cours d'alimentation (observation 1). La fibroscopie effectuée en urgence a alors permis l'extraction d'un corps étranger à type noyau de la pulpe de « pain de singe » (observation 1) et la découverte fortuite d'un anneau au niveau de la bouche œsophagienne de Killian dans les deux cas. Dans le SPV, le mécanisme de la dysphagie et la formation de la membrane ne sont toujours pas élucidés. Ainsi, même si la carence martiale n'est pas nécessaire à la formation de l'anneau, elle précéderait toujours la dysphagie [5]. Toutefois, la membrane ne serait pas pathognomonique du SPV. Elle ne serait pas en effet, nécessaire à l'apparition des troubles de la déglutition mais jouerait plutôt un rôle d'aggravation d'ordre mécanique [9]. L'association d'un SVP à une maladie de Biermer comme dans notre observation 2 est rarement décrite. Aldor [10] a été le premier à décrire un cas de SPV développé au cours du suivi d'une anémie pernicieuse. Peu après, un autre cas isolé de dysphagie par anneau de l’œsophage cervical sur Biermer était décrit par Procopis [11]. En Afrique au Sud du Sahara, le premier cas a été rapporté par Diop [3].

D'autre part une association entre SPV et syndrome de Goujerot-Sogrën a été décrite [12]. Ce dernier cas, celui de notre patiente, ajouté à ceux décrits par Procopis et Aldor ne plaident pas en faveur d'une association fortuite. L'argumentaire sur une origine auto-immune du SPV est ainsi fortement suggéré à nouveau. Mais la carence martiale due à une anémie pernicieuse semble être la cause la plus logique. L'expression hématologique habituelle du SPV est une anémie hypochrome microcytaire avec un abaissement de la ferritinémie. Cependant, dans nos observations la biologie avait mis en évidence respectivement une ferritinémie basse sans anémie et une hyperferritinémie avec anémie. En effet, la plupart des tests explorant le métabolisme du fer, permettent seulement d’établir l’état martial du malade à un moment donné. Aussi, avec le temps le taux de fer sérique augmente ou diminue de la même façon que les réserves en fer. Les mesures répétées donnent donc une estimation plus fiable des réserves totales en fer de l'organisme que les mesures isolées [13].

Le traitement est basé sur la supplémentation en fer. Celle-ci, entraine dans la majorité des cas une régression de la dysphagie avant même la normalisation biologique de l'anémie et malgré la persistance de l'anneau œsophagien [14]. Cependant, selon Jones [1] un anneau obstructif est souvent à l'origine d'une résistance au traitement rendant nécessaire une rupture endoscopique (ou des séances de dilatations par bougie) pour la régression de la dysphagie. L’évolution a été favorable chez nos patients après traitement martial, avec une nette amélioration clinico-biologique. Secondairement ils ont bénéficié d'une dilatation endoscopique. Toutefois, dans les séries de Neil [15] et Ben Dhaou [16], il a été noté des cas de cancer de l'hypopharynx chez des malades présentant un syndrome de Plummer-Vinson. Ainsi, même si un lien formel entre le cancer de l’œsophage et le SPV n'a pu être établi, nous recommandons une surveillance endoscopique au moins annuelle chez nos patients

## Conclusion

Malgré la fréquence de l'anémie ferriprive en Afrique, le syndrome de Plummer-Vinson y est encore peu rapporté. La dysphagie passagère, le jeune âge et le sexe masculin peuvent en faire errer le diagnostic. Son association avec une sténose caustique de l’œsophage et une maladie auto-immune est possible. Le risque accru de dégénérescence devrait motiver une surveillance endoscopique rapprochée.
